# Supplementary material for: CPF Induces GC2spd Cell Injury via ROS/AKT/Efcab6 Pathway
Source: Cells. 2025 Jun 20;14(13):940. doi: 10.3390/cells14130940 (PMC12248639; doi:10.3390/cells14130940)
Supplement: Supplementary file 1 [file cells-14-00940-s001.zip › cells-3636441-supplementary.pdf]

Caption tables and figure legends of supplementary file

Table S1 qRT-PCR reaction system

Table S2 qRT-PCR reaction program

Table S3 qRT-PCR reaction program

Figure S1 The protein expression of ferroptosis in GC2$_{spd}$ cells after CPF treatment for 24 hours. (A) Expression of FTH1; (B) Expression of CD71.

Figure S2 The detection of GC2$_{spd}$ cells after SC79 and CPF co-treatment for 24 hours. (A) Optimal concentration screening for SC79; (B) Phenotypes of GC2$_{spd}$ cells; (C) Cell viability of GC2$_{spd}$ cells. Scale bar indicates 100 μm.

Table S1 qRT-PCR reaction system

	12 μL
2X SYBR	6 μL
Forward primers	0.5 μL
Reverse primers	0.5 μL
cDNA（1/50）	5 μL

Table S2 qRT-PCR reaction program

Steps	Tm (^{o}C)	Times
1	95 ^{o}C	5 min
2	98 ^{o}C	10 s
3	60 ^{o}C	30 s
4	72 ^{o}C	30 s
5	go to 2	40 cycles
6	the dissolution of the curve	

Table S3 qRT-PCR reaction program

Antibodies	Companies	Cat
PI3K	Shenyang WanLei Biological Technology Co., LTD, China	WL03380
AKT	Shenyang WanLei Biological Technology Co., LTD, China	WL0003b
p-AKT	Shenyang WanLei Biological Technology Co., LTD, China	WLP001a
FTH1	AbMART, China	T55648
CD71	AbMART, China	T56618
beta Actin	Proteintech Group, Inc., China	60008-1-lg
HRP-conjugated Goat Anti-Rabbit IgG	InCellGenE LLC., America	SA-10011
HRP-conjugated Goat Anti-mouse IgG	InCellGenE LLC., America	SA-10010

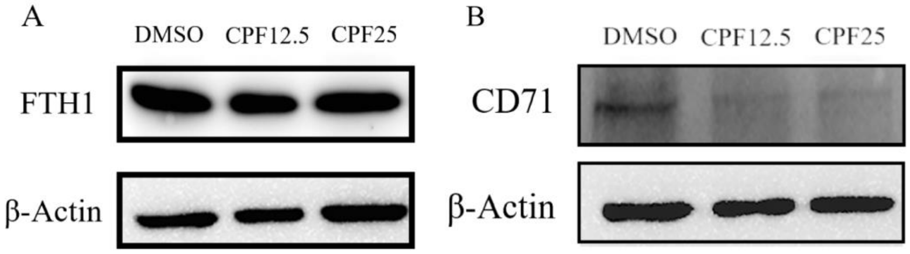

Figure S1 The protein expression of ferroptosis in $GC2_{spd}$ cells after CPF treatment for 24 hours. (A) Expression of FTH1; (B) Expression of CD71.

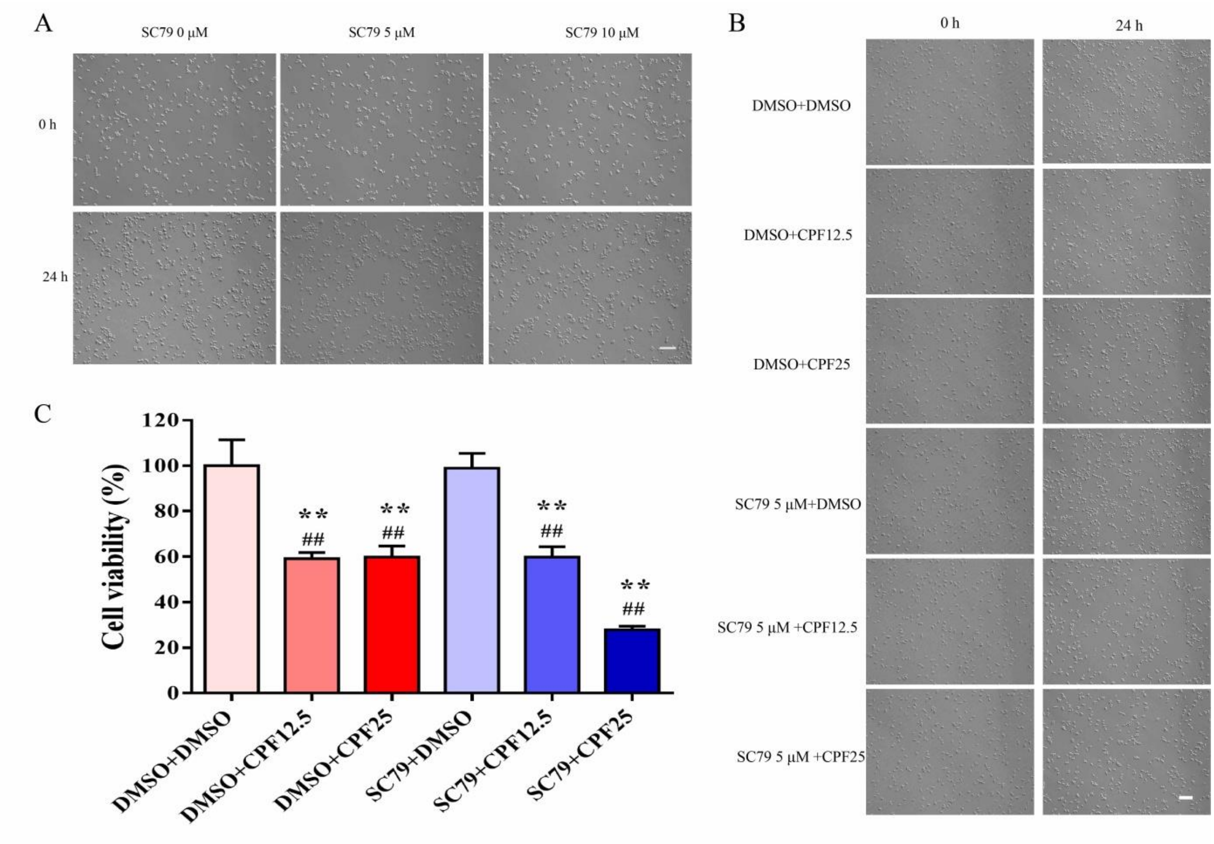

Figure S2 The detection of GC2$_{spd}$ cells after SC79 and CPF co-treatment for 24 hours. (A) Optimal concentration screening for SC79; (B) Phenotypes of GC2$_{spd}$ cells; (C) Cell viability of GC2$_{spd}$ cells. Scale bar indicates 100 μm.
